# Gesture, spatial cognition and the evolution of language

**DOI:** 10.1098/rstb.2021.0481

**Published:** 2023-04-24

**Authors:** Stephen C. Levinson

**Affiliations:** Max Planck Institute for Psycholinguistics, Nijmegen, 6525XD, The Netherlands

**Keywords:** evolution of language, interaction engine, gesture, spatial cognition, hippocampus, primate communication

## Abstract

Human communication displays a striking contrast between the diversity of languages and the universality of the principles underlying their use in conversation. Despite the importance of this interactional base, it is not obvious that it heavily imprints the structure of languages. However, a deep-time perspective suggests that early hominin communication was gestural, in line with all the other Hominidae. This gestural phase of early language development seems to have left its traces in the way in which spatial concepts, implemented in the hippocampus, provide organizing principles at the heart of grammar.

This article is part of a discussion meeting issue ‘Face2face: advancing the science of social interaction’.

## 1. The interaction engine: linguistic diversity versus constancy in the interactional base

There is no shortage of speculations about the origins of language (see [1] for an excellent review), but one neglected factor is the stark contrast between the diversity of languages on the one hand and the relative uniformity of the interactional structure in which they are embedded [2]. This contrast may offer some important clues to those origins in deep time.

There are about 7000 languages spoken by humans around the planet. Many of these are spoken by small, ethnic populations in the equatorial regions, and are in danger of being eased out by national pressures, migration, war and economic pressures. Conscious of this impending loss, recent efforts have been made to catalogue, preserve records and describe these languages^1^. These efforts have revealed that languages are far more diverse and varied than the linguistic theories of the last century had maintained: languages differ so strikingly at every level of organization, from the sound systems, to the morphology (word structure) and from the syntax to the semantics, that it is hard to find any non-trivial generalizations that hold exceptionlessly across all of them [6]. This makes the human communication system a biological curiosity—there is no other organism that has a communication system that varies so fundamentally at every level across social groups. It is true that other species with vocal learning may have distinctive dialects, but even at the sound level, the range of human language variation is of a different order: languages may use as many as 140 phonemes (distinctive sounds), or as few as 11, and they may employ very different parts of the vocal tract to make those sounds [7]. For example, located on two islands in the Coral Sea off Papua New Guinea, one language Yélî Dnye (on Rossel Island) has 90 phonemes [8], half of which are nasalized, while Rotokas (on Bougainville) has just 11, and none of them is contrastively nasalized: in the one language the velum (the end of the soft palate which can close off the nasal chamber) is doing velar gymnastics on a 10 ms timescale, and in the other it is a redundant organ. The more we learn about the languages of the world, the more impressed we should be by the extraordinary variety they exhibit. In addition to all these varieties of spoken language, in deaf communities the entire communication system is shifted with equally expressive potential from the vocal–auditory channel to the manual–visual one in some 300 sign languages which are almost as distinct from each other as spoken languages are.

Despite this impressive diversity of languages, there are some striking similarities in the ways in which they are used. The primary niche for language usage is in face to face interaction—this is the niche in which language is predominantly used, and where it is first learned by children. Extrapolating from a cross-cultural sample [9], it seems that on average we spend something like 3 h per day conversing, during which we may each produce 1500 utterances. A dispassionate observer looking across cultures would note many uniformities of the conduct of communication in this niche: humans huddle in small groups, orienting their bodies towards each other in a face-to-face pose, and one at a time exchange short bursts of communication of about 1–2 s duration on average, with a very rapid exchange of turns within a mode of 200 ms. Each burst of communication consists of a mini-performance, with a vocalization accompanied with gestures and facial expressions. This is a very distinctive ethology, and in general character it is more or less uniform across the species.

Underlying this distinctive communicative ethology is what has been called the ‘interaction engine’, a set of propensities and abilities that enable it [2]. Let us concentrate here on four of these shared properties: multimodality, turn-taking, contingency across turns, and inferences of communicative intent. First, in face-to-face conversation, each turn at speaking is accompanied by a multimodal display, with body position, gaze, facial expressions and manual gestures all deployed. There are intricate interconnections between the streams of signals sent out on these different articulators [10]. The speaker may start gazing at an addressee then look away, returning at the end of the turn [11]. During the phase of mutual gaze, the speaker seems to adjust the length of the turn according to the blink durations of the addressee [12]. The white sclera of the human eye makes these fine signals available to addressees and is likely an evolutionary adaptation to this interactional niche. Most utterances are accompanied by manual gestures, some of which carry semantic content, while others emphasize prosodic structure and both of which can play a role in signalling turn transitions [11]. It is noticeable that any kind of spatial description (involving layout, shape, motion, etc.) is likely to be accompanied by gesture. Meanwhile facial expressions are used by both speaker and addressee to signal attitudes relevant to the utterance. How all these streams of behaviour packaged into these bite-sized bursts of communication are initiated, controlled, synchronized and comprehended remains relatively unexplored. As far as we know, barring special social conventions and taboos (like gesturing with the left hand in West Africa [13], or gazing too intently in Mayan societies [14]), this multimodal deployment looks very similar across language after language.

Fast turn-taking is a central property of conversation. As mentioned, turns are on average just under 2 s long, and the modal gap between turns is, depending a bit on the sample, around 200 ms. Two hundred milliseconds approximates the speed of the fastest human response time to an expected signal, so turn-taking is at the extremes of human performance. Even more suprising is that this rapid response can be achieved despite the fact that the latency for word retrieval and speech encoding is upwards of 600 ms—in practice to encode a sentence from scratch will take well over a second [15,16]. The only way that this fast turn-taking can in fact be achieved is by partially switching from comprehension to sentence production mid-way through the incoming turn, with a distinct neural signature for the switchover [17,18]. This is a cognitively intensive form of double-tasking, partly using the same neural pathways for output and input. These patterns of turn-taking are very similar across languages and cultures. In a study of conversation in ten languages, there were differences in mean timing, but they were all in the same sort of ballpark, within 200 ms of a cross-linguistic mode [19]. Most telling, perhaps, is the pattern in sign languages, where the same 200 ms modal response time has been found [20], despite the completely different channels involved.

A third important property of communication in the interactional niche is the contingency that holds across turns: questions mostly get answers, requests compliances, offers acceptances or rejections, greetings, etc. (so-called ‘adjacency pairs’, see [21]). The contingencies are of a different order of complexity from those found in other animal communication systems. First, there is a huge (even indefinite) range of speech acts (or social actions), i.e. the types of the utterances (e.g. a question about X) that require or prefer corresponding responses (an answer about X). Second, there is an ordered set of possible responses, where the preferred response is fast, the dispreferred slow [22]. Third, the contingencies can be used to build large structures organizing interaction, as in the following exchange, which has a question–answer pair embedded within a question–answer pair:

A: ‘May I have a bottle of Mich?’
B: ‘Are you twenty-one?’
A: ‘No’
B: ‘No’.

This is what linguists have called centre-embedding, a central case of recursive structure. Interestingly, these structures can be found six deep in discourse, which is far deeper than anything found in syntax where it has been claimed recursive structures originate [23]. Structures of this kind can be embellished, with a lead-in contingent pair (e.g. ‘Hey barman!’ ‘What?’), and a lead-out contingent pair ‘Oh’ ‘Sorry’), and so forth. There are many intricate details here, and they have been shown to pattern more or less identically across a dozen unrelated languages [24]. Similarly, there are contingency structures for repair, when an utterance has not been heard or understood. These structures again appear to be universal across languages and cultures [25], with even the repair initiator ‘huh?’ having similar form and function across most languages [26].

A fourth essential feature of the ‘interaction engine’ is the attribution of intent to utterances, requiring the ability (involving what has been called ‘theory of mind’) to model the speaker's communicative goal in order to aid comprehension (see [27]). This is a crucial property because linguistic structures are never fully determined—they require disambiguation and contextual resolution, as fits the direction of the talk. An utterance like ‘Making a new vaccine will take some time’ is a truism, but in context will have a definite interpretation; ‘Don't forget to call what's his name’ presumes the addressee can figure out who the speaker had in mind; ‘Well done!’ will not be congratulations in response to ‘I've lost the front door key’. More generally, because of finite vocabularies, inevitable vagueness and ambiguities, and the fact that the speech act (the main point) is often not coded, few utterances fully specify the intended message. Consider the exchange:

A: ‘I could eat the whole of that cake!’
B: ‘Oh thanks!’,

where nothing in A's utterance indicates directly it is a compliment, as B presumes. This kind of intention attribution is vital to the workings of human interaction, and along with the celebrated recursive nature of syntax gives language its indefinitely large communicative resources.

These properties appear to be strongly universal, in the sense that human communicative interaction in every language and culture appears to exhibit them. They are also apparent in the absence of a shared language, most notably in the phenomenon of ‘home sign’ when a deaf child is born into a hearing family with no available institutional sign language and invents a gesture system of its own in conjunction with its caregivers [28]. Many of the properties are also visible in infant proto-conversation, where a pre-linguistic infant exchanges multimodal signals non-verbally with its caregivers. These properties are also shared in quite large part with the other members of the great ape family [27]. This seems to indicate that the interaction engine is part of a deep ethological substrate to human communication.

## 2. A puzzle: language structure appears remarkably independent of the interactional niche

Given the robust constancy of the interactional niche across languages and the fact that it is where the bulk of language use occurs and where children learn language, one might have expected there to be many deep ways in which languages are adapted via cultural evolution to the context where they are so intensively used. But in fact such stigmata of the interactional niche are far from obvious. For example, given the universal and intensive character of turn-taking, one might have expected there to be clear ‘over and out’ signals of the kind used on two-way radios—instead, the ends of turns are judged by a complex and distributed set of features [11]. Or, given the multimodal nature of that niche, one might have expected an elaborate syntax that could freely embed non-vocal signals sequentially along with vocal ones. This occurs only to a limited extent, as in ‘The two cars went [claps hands]’ (see [29]). Instead, multimodal signals are normally layered over one another (as when saying ‘This one’ with simultaneous pointing). On the face of it then, linguistic structure looks remarkably independent of the interactional niche, and this has made it possible for linguists like Chomsky [30] to claim that language did not evolve for communication at all.

Here I propose two responses. The first is to point out that there are in fact many ways in which natural languages adapt through cultural evolution to the interactional niche, certainly more than normally meet the linguist's eye. This section spells those out. The second is to sketch how in fact the very core of language structure may owe its origin to the multimodal niche in which language most likely evolved, which is the subject of the rest of the paper.

It is possible to make quite a list of features of linguistic structure and organization that indubitably owe their origin to the interactional niche. Consider for example the so-called McGurk effect, wherein people hearing ‘ba’ but seeing lips moving as if for ‘ga’ hear an intermediary sound, often ‘da’ [31]. The phenomenon illustrates the confluence of auditory and visual information experienced in the interactional niche. In a similar way, it has been shown that mismatches between gesture and speech confuse the understanding of narrative [32]. Another telling detail is that normal speech levels (*ca* 65 dB) are clearest in a 1–4 m range, the typical interactional huddle, while the critical 2–4 kHz band associated with fine consonant discrimination is most intelligable at a face-to-face distance of just 1 m.

Consider now that the basic grammatical unit, the minimal clause, seems temporally fitted to the typical turn length of just under 2 s. This is not surprising, as the first part of a contingency pair (a question, request, offer or the like) is normally a full clause, while the response may be more elliptical. The function of such a turn, of course, is basically to deliver a speech act, and some speech acts like questions, requests, proposals, etc. come with language-specific grammatical marking (interrogative, imperative, optative marking and the like), although many do not [33].

Another notable adaptation of language to the interactional niche is deixis, namely the use of words that point to features of the niche—‘you’ indicates the addressee, ‘here now’ the place and time of speaking, so grounding person, tense and place in the current interchange. Moreover, ‘this finger’ or ‘over there’ or ‘not you, but you’ provide slots for gestural specification, so exploiting the multimodality of the niche.

Linguists have also noted that many constructions that re-order elements of clauses have interactional motivations. For example, instead of saying 'I seem to remember the last paragraph', the object can be fronted in order to assure correct identification before proceeding (after [34, p. 24]):

A: 'The last paragraph'
B: 'Yes'
A: 'Em, I seem to remember it being different from what's printed'.

In addition, as pointed out in [35], syntactic dependencies can hold across turns, as when one speaker completes the utterance of another.

There are many properties of the *use* of language that are tailored to the interactional niche. For example, the rapidity of turn-taking makes it useful to use fillers (like 'em') to buy time, and the overlap of comprehension and production towards the end of the turn makes it advantageous to ‘front load’ a turn so that its function is visible as early as possible [33]. Yet despite all this, the core of linguistic structure, the structural relations between nouns and verbs (or the concepts they denote) for example, seems remarkably independent of the niche in which language is so intensively used. So our puzzle about the apparent disconnect remains. We turn now to an alternative line of enquiry, which seeks to trace the heart of linguistic structure to the interactional niche through a deep evolutionary connection between spatial cognition and language.

## 3. Spatial cognition in language structure

There is a long tradition in the analysis of language, often called localism, going right back to Aristotle that holds that spatial concepts play a central role in structuring grammar and semantics [36]. For example, the German philologists of the nineteenth century observed that nearly all the grammatical cases in Greek, Latin and Sanskrit had spatial origins, meaning ‘to’, ‘from’, ‘at’ and so on, before becoming generalized into abstract grammatical cases like datives, accusatives and genitives. Cases (or analogous constructions with prepositions) play a central role in language because the heart of the grammar of any language is the way noun phrases are bound to verbs, and this is precisely what cases and prepositions (or postpositions) do. The binding of nouns to a verb by specific relations (the actor, the theme or thing acted on, the instrument used, and so on) is what expresses a proposition, a statement about the world. In the twentieth century earlier localist ideas were exploited to construct case-based models of grammar [37,38], ideas that persist in modern linguistic theories.

Although modern English has no cases, these processes can be seen in the development from Old English affix ***æf*** ‘away from’ into the genitive preposition as in ‘The Bishop of Liverpool’, and then into an abstract binder of nouns to verbs as in ‘I've heard *of* it’, ‘it's made *of* wood’, ‘it smells *of* teak’. One can observe a systematic transfer of meaning from the spatial domain to other domains as sketched in table 1. The observation that non-spatial relations evolve from spatial ones was generalized into theories of semantics, on the grounds that spatial cognition plays a central role in human cognition generally (e.g. [40–42]). Thus spatial expressions are used to describe temporal relations (*from now on, at noon, on Wednesday*) but also many evaluative domains (*a low price, top talent, beneath contempt*). Linguists noted the complex patterns of extensions of meaning from out of the spatial domain not only in language change [43] but also in usage and metaphor [44].

**Table 1.** Transfer of spatial concepts across domains (after [39]).

space	possession	properties	schedules
‘he's in Paris’	‘it's in his possession’	‘he's in a depression’	‘it's in a week’
‘he went to Paris’	‘the house went to him’	‘the light went to green’	‘it's changed to Tuesday’
‘he kept it there’	‘he kept the money’	‘it was kept green’	‘it's been kept to Tuesday’

A complete model of semantics built on spatial concepts was developed in Jackendoff [41], which included as atoms of meaning *places* and *paths* (to, from, away, etc.), so states could be represented as things in places (*He's in trouble*) and events as motions of things along paths (*The store went bust*). The representation of *John entered the room* is then something like [_event_ GO [_thing_ JOHN], [_path_ TO [_place_ IN [_thing_ Room]]]].

In short, the theory of localism has a long history, and is supported by many observations about language structure and change, and many of its insights have been incorporated into contemporary theories of language. The theory suggests that spatial concepts play a critical role at the core of language structure.

## 4. Spatial cognition in the brain

O'Keefe & Nadel [45] suggested more than 40 years ago that the hippocampus is the seat of mammalian cognitive maps. After years of careful experimentation using direct cell recordings of the rat brain, O'Keefe and associates have uncovered the neural mechanisms involved in establishing location, orientation and way-finding. Specialized cells were discovered in the hippocampus and entorhinal regions that fire just when the rat is in a location (place cells) embedded in a map of locations (grid cells of different resolution), or is near an orienting location (boundary cells); additional head direction cells and speed cells (measuring distance over time) were also discovered. Using some equivalence of vector addition or substraction, the rat is able to extrapolate how to get from A to B, and then back to A via C. The work established one of the most direct implementations ever discovered of a higher cognitive function in its neural substrate.

The same mechanisms have been found operative in humans. The right hippocampus continues to do what it does in rats, and has been shown to actually grow as a taxi driver learns new routes [46]. The mental maps in the right hippocampus drive the gestures that we make when giving route directions and other spatial descriptions [47]. But in humans language has partly altered the picture by co-opting the left hippocampus for the purposes of linguistic computation and verbal memory. The evidence for this has been slowly accumulating. When you learn a new language, this hippocampus increases in grey matter volume just like the right hippocampus does when learning new routes [48]. It can also be shown to track reference to actors in narratives [49,50]. In a parallel to spatial navigation, where one has expectations of what lies beyond a familiar corner, so the hippocampus tracks expectations about how sentences will finish. So, for example, direct cell recordings that parallel those in the rat show stronger theta-rhythm for sentences with predictable endings (like *He locked the door with a …*) than unpredictable ones (like *She came in here with a …*)—the same rhythm that reflects the rat's prediction of direction [51,52].

This co-option of the left hippocampus by language was already hypothesized by O'Keefe & Nadel [45]. O'Keefe [53,54] has consistently pursued the hypothesis, offering developed sketches of how the neural implementation of space in the hippocampus offers a framework for linguistic structure in ‘vector grammar’. He has argued that the inventory of specialized cells (place cells, boundary cells, head direction cells, etc.) provides a framework of vectors that can accurately model spatial descriptions. So for example just as the rat's boundary cells fire more in increasing proximity in the direction of the expected boundary, making an oriented tear-drop-shaped field, so an English speaker's estimations of whether X can be said to be *under Y* show a similarly shaped field in a vertical direction beneath a reference object [54]. A number of directional vectors may be involved, as with complex spatial notions like *between.* The intuition here is that the inventory of cell types and their vector interconnections establish a rich network capable of supporting elaborate semantic specifications. The cell inventory indeed bears a close resemblance to the inventory of spatial primitives proposed by Jackendoff [41] reviewed in §3 above. In both cases it is necessary to add a temporal and a causal dimension, as both O'Keefe and Jackendoff presume. This is a compelling convergence from cell biology with the independently derived linguistic theories.

One interesting possibility is that the co-option of the left hippocampus for linguistic purposes rather than spatial cognition may actually have impaired human native spatial abilities. Studies of Western urban populations, who tend to use an ego-centric (left/right/in front/behind) mode of wayfinding, show much lower abilities to maintain a sense of direction during path integration than many other animals [55]. There is evidence that extensive wayfinding experience associated with hippocampal growth may come at the cost of other abilities [56], so the inverse pattern is likely. Humans however have developed elaborate spatial prostheses—compasses, maps, navigational aids—to compensate for lost wayfinding abilities. Interestingly, one of these cultural substitutions for lost native abilities is a kind of special linguistic and gestural system: populations who use an exclusively cardinal-direction system in language and gesture evince a much higher ability to do path integration and maintain a sense of direction, because the communication system mandates constant updating of orientation [55].

## 5. Gesture as the Trojan horse: how spatial cognition came to structure language

Although the thesis of localism has been entertained by many, the question of why language came to co-opt the hippocampus in particular has not been much addressed. O'Keefe & Nadel [45] suggest in a footnote that it may have been because our early forebears wished to communicate foraging directions. However, enquiries about the evolutionary origin of the ‘interaction engine’ suggest another route. In every major branch of the primate order at least some species can be found that are vocal turn-takers [57]. But in the Hominidae, the family of great apes, of the eight species normally recognized (3 orangutan species, 2 gorilla species, 2 chimpanzee species and humans), only humans are vocal turn-takers. This is because the flexible, negotiating communication system of the non-human apes is primarily gestural, and they lack the fine breath control, and the correlated audiograms, that are prerequisites for speech [58]. It should be pointed out though that, as with humans, ape gestural communication is also multimodal, involving facial expression and occasional vocalization accompanying gesture [59]. An interesting finding is that for at least some of these species, the timing of turn-taking has the same fast response speed (*ca* 200 ms) found in human vocal exchanges [60–62]. The great apes also seem to share other features of the ‘interaction engine’, for example, some of the contingency and repair structures mentioned in §1 above [63].

**Figure 1.**
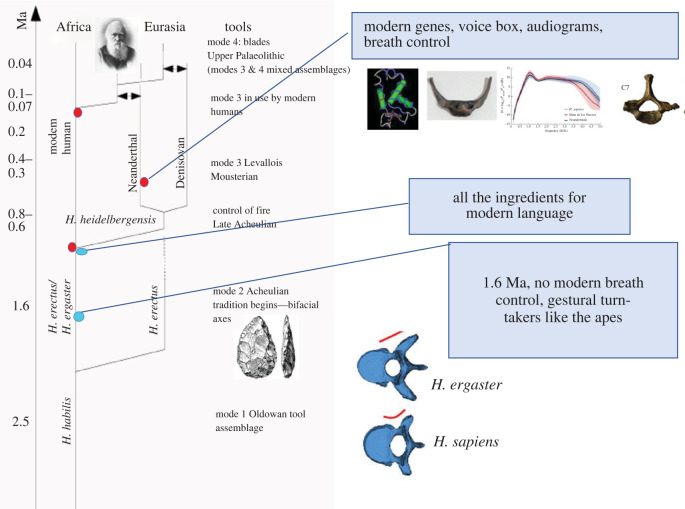
Landmarks in the evolution of language (after [64,65]). On the timing of the gestural-to-vocal transition see Dediu & Levinson [64,65] and Levinson & Holler 2014 [66]; on early auditory adaptations see Conde-Valverde *et al.* [67]; on early breathing adaptations see MacLarnon & Hewitt [68] and Bastir *et al.* [69]. (Online version in colour.)

Now given that all the great apes except humans are primarily gestural communicators in close interaction, it follows by the normal phylogenetic reasoning that the common ancestor was the same. The last common ancestor between humans and chimpanzees, living perhaps 6 Ma, was therefore likely a gesturer, as would have been the first hominins in our line. We now have much data pointing to the slow shifting of the primary mode of communication over the last 2 million years. Dediu & Levinson [64,65] read this evidence as follows (figure 1). Before 1.6 Ma, a species or species-cluster known as *Homo erectus* had begun colonizing much of the Old World. We have just one well-preserved vertebral column from this time period from the African version of *H.* *erectus*, often designated *H. ergaster.* This fossil (KNM-WT 15000) has a narrow vertebral canal in the thoracic region, similar to chimpanzees, while later hominins have a broader canal which accomodates extra thoracic enervation for the fine control of breathing [70]. Voluntary breath control plays a fundamentally important role in speech, not just for powering vocalization, but also because it drives the intensity variations in every syllable. Although some scholars have wondered if this individual was pathological, the balance of opinion seems to be against that interpretation [69]. On that reading, this individual alive 1.6 Ma was primarily a gesturer. Interestingly, endocasts from a wide range of *H. erectus* crania suggest the development of language-related cortical areas in the period immediately after this, with the expansion of the prefrontal cortex (and the language-critical Broca's area) pushing back the precentral inferior sulcus [71]. If we go forward a million years we come to the branching between anatomically modern humans and Neanderthals. There is now a plethora of information about Neanderthals: they exhibit the genes known to be critical for language and speech, the wide thoracic vertebral canal essential for fine breath control, and a modern-like hyoid bone that sits above the larynx (see [64,65] for additional references). Moreover, audiograms recoverable from the bones of the middle ear show that proto-Neanderthal audition was already tuned to the bandwidth of modern speech [67]. It seems inescapable that Neanderthals were an articulate species. If so, we can project modern speech and language capacities back to the common ancestor between anatomically modern humans and Neanderthals, at perhaps 600 000 years ago. So somewhere between 1.6 Ma and 600 000 years ago the burden of communication was being increasingly loaded off gesture and onto speech.

**Figure 2.**
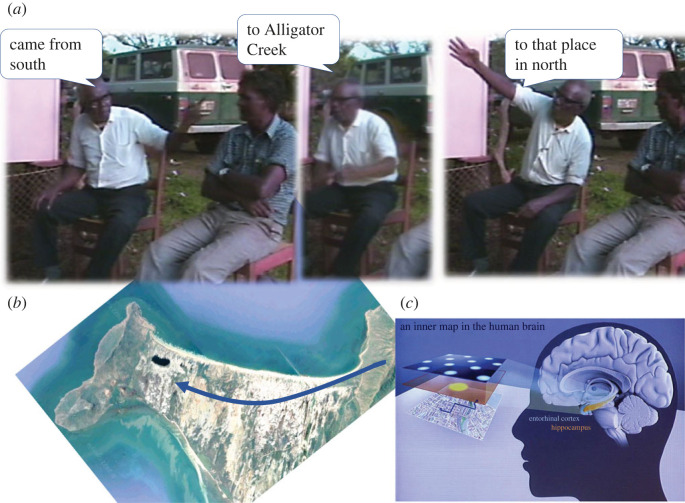
Gestures driven directly off the cognitive map. (*a*) Film sequence of a single Guugu Yimithirr sentence *gadaa* *dyibarra* *Alligator Creek nhayungu gunggaarr* 'You came from the south via Alligator Creek to that place in the far north'. (*b*) Route traced on a satellite image (rotated to match camera angle in (*a*)). (*c*) Graphic (taken from [73]) illustrating O'Keefe's Nobel Lecture [74]. (Online version in colour.)

But during the long gestation of spoken language, gesture was clearly the prime starting point. Gesture is a mode of communication that uses movements in space largely to indicate spatial content, that is, the location of things in the environment and the directions and manners of motion. In face-to-face communication, gesture is more or less obligate in spatial description—just try describing the layout of your home without gesturing. Nearly every turn at talk is associated with a gesture, at a rate of one every 2.5 s [72]. Spatial content anyway pervades speech: a fifth of the most common English words are spatial. This is especially true of the conversation of modern hunter–gatherers, who offer the best insight into our foraging ancestors. For example, Guugu Yimithirr speakers in North Queensland use north–south–east–west words to the exclusion of left–right–front–back vocabulary. About one in ten words in Guugu Yimithirr conversation is one of these cardinal direction terms, which divide the horizon into four quadrants. But the words only give 90° angles, so they are supplemented by gestures, and these gestures have a directional veracity to a few degrees of arc. You can directly relate the gestures to a survey map, and it is clear that the gestures are driven directly from a hippocampal cognitive map (figure 2; [55,75,76]). This gives some insight into the functional utility of the gestural channel in early prehistory. That impression is reinforced by examining the gestures used in ‘home sign’. ‘Home sign’ is a system of gestures that emerges spontaneously from a deaf child born to hearing parents, where there is no conventional sign language available. Studies show that there are striking parallels between independently invented gesture systems of this sort [28]. Spatial gestures—pointings to places and persons, action mimicry, shape outlines, etc. play a crucial role in getting these systems off the ground. I have studied an isolated deaf adult on Rossel Island who is able to play a fairly full role in social life through a gesture system that is only partly shared with those around him. As long as his narratives are spatially grounded, those familiar with him can follow quite abstract trains of thought (like sorcery accusations directed at particular individuals for allegedly causing the deaths of other individuals).

So the thesis here advanced is that gesture is the Trojan horse that imported spatial cognition into the heart of language. If early hominin communication had the burden of communication in the gestural channel, gesture would have inevitably drawn on the spatial expertise and organization in the hippocampus. As we gradually shifted the burden to the vocal channel, the underlying conceptual framework would have remained spatial. In this way, spoken language has preserved earlier stages of the multimodal interactional niche deep at the heart of linguistic structure.

This is of course a ‘gesture-first’ theory of language evolution [1]. Such theories dove-tail with a number of primary observations: Tool-using seems to have early used the hands freed by bipedalism, and the development of right-handed skills utilizes much of the language areas in the left hemisphere [77]. To this day, gesture activates a superset of the language networks in the brain, and hippocampal damage leads to gesture impairment [47,78]. The very persistence into modern times of a (largely) unconscious gesture system accompanying speech with intricate temporal alignment to it argues for an early dependence of speech on gesture and multimodality. Above all, the extraordinary human ability to switch entirely out of the vocal–auditory channel into the visual–manual one as in sign languages indicates continuing availability of the gestural modality. Proponents of gesture-first theories also point to mirror neurons that selectively fire both on visually perceived and directly enacted gestures, so priming input–output equivalence of the kind found in vocal learning [79].

Strong arguments against a gestural protolanguage have also been rehearsed. McNeill [80] has insisted rightly that modern linguistic communication is actually heavily multimodal, and the same holds for ape gestural communication, which co-occurs with facial expressions and vocalizations [59]. Thus any ‘gesture-first’ theory is actually only a theory about shifting the main informational burden from one modality towards another. A much more serious problem is raised by Emmorey [81], Levelt [82] and Fitch [1]: sign languages are such effective and sufficient modes of communication that once humans had got into that deep evolutionary valley, there would have been no way out of it—no fitness incentives would have been sufficient to shift modalities. A possible response here is that perhaps a gestural proto-language never approached the effective sign language stage, remaining more like the ad hoc gestural systems seen in ‘home sign’, before slowly beginning to shift the burden across the multimodal channels toward speech. Moreover, the positive virtues of speech (broadcast communication at a distance and in the dark) and better still, a multimodal system, must have had a countervailing influence.

## 6. Summary and conclusion

The interaction engine provides a stable cross-cultural base for the use and acquisition of language, with hallmarks shared with our great ape cousins, including its rich deployment of multimodal signals. This ancient primate heritage is reflected in our continued use of gesture. By contrast, vocal language seems a relatively late overlay that evolved in the last one million years. Unlike the interaction engine, vocal language transmission has been largely outsourced to culture, with the consequent diversity of languages. Although linguistic structure apparently shows a remarkable independence from the interactional system that is its predominant ecological niche, that niche has likely left its multimodal signature deep in the heart of language structure, through the import by gesture of spatial cognition into our communication system.

## Data Availability

This article has no additional data.

## Footnotes

1 Online databases used include Glottolog 4.5 (https://glottolog.org/), WALS [3], FOIBLE 2 [4] and GRAMBANK [5].
